# A Rolling Element Bearing Fault Diagnosis Approach Based on Multifractal Theory and Gray Relation Theory

**DOI:** 10.1371/journal.pone.0167587

**Published:** 2016-12-30

**Authors:** Jingchao Li, Yunpeng Cao, Yulong Ying, Shuying Li

**Affiliations:** 1 College of Electronic and Information Engineering, Shanghai Dianji University, Shanghai, China; 2 College of Power and Energy Engineering, Harbin Engineering University, Harbin, China; 3 School of Energy and Mechanical Engineering, Shanghai University of Electric Power, Shanghai, China; 4 Shanghai Electric Gas Turbine Co., Ltd., Shanghai, China; Chongqing University, CHINA

## Abstract

Bearing failure is one of the dominant causes of failure and breakdowns in rotating machinery, leading to huge economic loss. Aiming at the nonstationary and nonlinear characteristics of bearing vibration signals as well as the complexity of condition-indicating information distribution in the signals, a novel rolling element bearing fault diagnosis method based on multifractal theory and gray relation theory was proposed in the paper. Firstly, a generalized multifractal dimension algorithm was developed to extract the characteristic vectors of fault features from the bearing vibration signals, which can offer more meaningful and distinguishing information reflecting different bearing health status in comparison with conventional single fractal dimension. After feature extraction by multifractal dimensions, an adaptive gray relation algorithm was applied to implement an automated bearing fault pattern recognition. The experimental results show that the proposed method can identify various bearing fault types as well as severities effectively and accurately.

## 1. Introduction

As a main component in almost all types of rotating machine rolling element bearings have been used widely. Bearing failure is one of the main causes of failure and breakdowns in rotating machinery, leading to significant economic loss [1–3]. So as to keep machinery running with high reliability and availability, it is necessary to develop a reliable and effective bearing fault diagnosis method. Among various bearing fault diagnosis methods, vibration-based analysis methods have arrested extensive attention in the last few decades [4]. Bearing vibration signals contain rich information about machine health status, and thus it is possible to obtain foremost characteristics from vibration signals by signal processing techniques [5]. Nowadays a great number of signal processing methods have been used for rolling bearing fault detection and diagnosis. However, due to the nonlinear factors, e.g., friction, stiffness and clearance, bearing vibration signals in particular under faulty conditions, will behave nonstationary and nonlinear characteristics [6]. In addition, the measured vibration signals contain not only the working condition information concerning the rolling bearing itself, but abundant information of other moving parts and structures of machinery, which belongs to the category of background noise compared with the former [7]. As background noise is often comparatively large, the slight bearing fault information may easily be submerged in the background noise and become hard to extract and detect. Therefore, the conventional time and frequency domain methods which aim mainly at linear vibration signals, and even advanced signal processing methods (e.g., wavelet transform) may not make an accurate assessment of the rolling bearing health status [8].

With the development of nonlinear dynamics theory, a great deal of nonlinear analysis techniques have been proposed aiming at recognizing and predicting the complex nonlinear dynamic behavior of bearings [4,9]. One of the most common manners is to refine and extract the fault features from vibration signals by combination of a few of advanced signal processing methods (e.g., wavelet package transform (WPT) [8,10], hilbert transform(HT) [10,11], empirical mode decomposition (EMD) [11] and higher order spectra (HOS) [12]), to recognize the fault frequency used to compare with the theoretical value with involvement of expert’s empirical judgement. With the advent of artificial intelligence, the process of rolling bearing fault diagnosis is more and more treated as a procedure of fault pattern recognition, and its effectiveness and reliability significantly depend on the selection of dominant characteristic vector of the fault features [13]. Recently, Some entropy based methods, e.g., approximate entropy (ApEn) [14,15], sample entropy (SampEn) [16], fuzzy entropy (FuzzyEn) [16,17], hierarchical entropy (HE) [13,18] and hierarchical fuzzy entropy (HFE) [13], have been proposed to to extract characteristic vector of the fault features from bearing vibration signals. In the paper, we introduce a multifractal theory based method, i.e., a generalized multifractal dimension algorithm, to extract dominant characteristic vector of the fault features from bearing vibration signals. Fractal theory is one of the most important branches for the contemporary nonlinear science, and is in particular suitable for processing all kinds of complex nonlinear and nonstationary phenomenon [19] and thus may also suitable for fault feature extraction from bearing vibration signals.

Usually, after feature extraction, a pattern recognition technique is needed to achieve the rolling element bearing fault diagnosis automatically [13]. A series of pattern recognition methods have been introduced for mechanical fault diagnosis nowadays, among which the most commonly used ones are artificial neural networks (ANNs) [20,21,22] and support vector machines (SVMs) [23]. The training of ANNs always needs a large number of samples which may be difficult or even impossible to achieve in practical applications, especially for the fault ones. While SVMs based on statistical learning theory, which is of specialties for a smaller number of samples, have better generalization than ANNs and ensure that the local and global optimal solution are exactly the same [24]. However, the accuracy of a support vector machine (SVM) classifier is highly decided by the selection of optimal parameters for SVMs [24,25]. To ensure the diagnostic accuracy, an optimization algorithm [24,25] or / and complex multi-class concept [13,26] have to be used subsidiarily to improve the effective of SVMs. Here, in order to solve the issue of generality versus accuracy, an adaptive gray relation algorithm was developed to achieve accurate patter recognition based on a small number of samples.

In summary, a novel approach for rolling element bearing fault diagnosis was proposed based on multifractal theory and gray relation theory. First, the fault features from bearing vibration signals, which offer more useful and distinguishing information imaging different bearing health status were extracted by generalized multifractal dimension algorithm. And then different fault types of rolling element bearing as well as severities were recognized by an adaptive gray relation algorithm. The rest of this paper is organized as follows. A review of conventional single fractal dimension and its improved version is introduced separately in section 2.1 and 2.2. The gray relation theory for fault pattern recognition is given briefly in section 3, followed by section 4 which presents the proposed bearing fault diagnosis method. Next, experimental validation of the proposed method is given in section 5.1 and 5.2. Finally, conclusions are illustrated in section 6.

## 2. Fractal theory

Fractal theory is one of the most important branches for the contemporary nonlinear science, and is a novel scientific method especially suitable for processing various complex, nonlinear, irregular and nonstationary phenomena [27], and thereafter may be also suitable for fault feature extraction from bearing vibration signals.

### 2.1 Traditional single fractal dimension

Fractal theory has been used to radar signal analysis [27], in which fractal dimensions such as box-counting dimension, Higuchi dimension, Petrosian dimension, Katz dimension, Sevcik dimension and so forth [28], are applied to depict the complexity of radar emitter signals. And among them, box-counting dimension is one of the most common fractal dimensions due to simplicity of its algorithm, and its algorithm is described as follows.

Given that *A* is a nonempty bounded subset of Euclidean space *R*^*n*^ to be calculated, and *N*(*A*,*ε*) is the least number of boxes covering *A* with the side length of *ε*, and therefore the box-counting dimension can be defined as:

$$D=\underset{\varepsilon \to 0}{\lim}\frac{\log N(A,\varepsilon )}{\log(1/\varepsilon )}$$ (1)

For the actual bearing vibration discrete signals, due to existence of the sampling frequency, the sampling interval *σ* is the highest resolution for the signals. Therefore, it is no meaning to calculate the box-counting dimension when *ε* → 0 and the minimum length of the box is often taken as *ε* = *σ*.

Considering a sequence of vibration discrete signals *x*(*i*) as a closed set of Euclidean space *R*^*n*^, the computational procedure of box-counting dimension is described as follows:

Adopt approximate method to make the minimum side length of the boxes covering the sequence of vibration discrete signals *x*(*i*) equal to the sampling interval *σ*, and then calculate the least number of boxes *N*_*kσ*_ with side length of *kσ* covering the sequence of vibration discrete signals *x*(*i*), thus:

$$p_{1}=\max\left\{x_{k\left(i-1\right)+1},x_{k\left(i-1\right)+2},\cdots x_{k\left(i-1\right)+k+1}\right\}$$ (2)

$$p_{2}=\min\left\{x_{k\left(i-1\right)+1},x_{k\left(i-1\right)+2},\cdots x_{k\left(i-1\right)+k+1}\right\}$$ (3)

$$p\left(k\varepsilon \right)=\sum_{i=1}^{N_{0}/k}\left|p_{1}-p_{2}\right|$$ (4)

where *i* = 1,2,⋯,*N*_0_/*k*, *k* = 1,2,…,*K*. *N*_0_ is the number of sampling points, *K* < *N*_0_. *p*(*kε*) is the longitudinal coordinate scale of the sequence of vibration discrete signals *x*(*i*). Therefore *N*_*kε*_ can be expressed as:

$$N_{k\varepsilon }=p\left(k\varepsilon \right)/k\varepsilon +1$$ (5)

Select a good linearity of the fitting curve log *kε* ∼ log *N*_*kε*_ as the scale-free area, thus:

$$\log N_{k\varepsilon }=d_{B}\log k\varepsilon +b$$ (6)

where *k*_1_ ≤ *k* ≤ *k*_2_, *k*_1_ and *k*_2_ are the beginning and end of the scale-free area respectively.

Usually, the least square method can be used to calculate the slope of the current fitting curve which is the fractal box-counting dimension *D* of the sequence of vibration discrete signals *x*(*i*):

$$D=-\frac{\left(k_{2}-k_{1}+1\right)\sum \left(\log k\right)\cdot \log N_{k\varepsilon }-\sum \left(\log k\right)\cdot \sum \log N_{k\varepsilon }}{\left(k_{2}-k_{1}+1\right)\sum \log^{2}k-{\left(\sum \log k\right)}^{2}}$$ (7)

However, single fractal dimension is often not enough to describe a complicated fractal object, and thus multifractal theory is introduced to analyze bearing vibration signals. Multifractal dimensions are the extension of conventional single fractal dimension, which can be applied to describe growth features of a fractal object at different levels, to compensate the lack of conventional single fractal dimension.

### 2.2 Generalized multifractal dimensions

The traditional single fractal dimension has been introduced in section 2.1, and has been widespreadly used in strictly self-similar signals such as the signals in the biological medicine, image analysis and electromagnetic fault diagnosis. Nowadays, single fractal dimension has also been used to quantitatively extract fault features from bearing vibration signals, which has attracted extensive attention from investigators. However, the common bearing vibration signals do not satisfy the self-similar structure of fractal theory to some degree. Therefore, when using the fractal box-counting dimension to calculate box-counting dimension of the vibration signals, the curve fitting often do not have good linear structure. Moreover, single fractal dimension only images the whole characteristics of vibration signals and lacks the depiction of local singularity of the vibration signals. In comparison, Multifractal dimensions are the extension of conventional single fractal dimension, which can be applied to describe growth features of the fractal object at various levels, to compensate the lack of conventional single fractal dimension. And the detail is described as follows.

Divide the research object (i.e., bearing vibration signals) into *N* small areas, and consider the linear dimension of the *i*_*th*_ area is *ε*_*i*_, and thus the probability density function *P*_*i*_ for the *i*_*th*_ area with different scale index *α*_*i*_ can be described as:

$$P_{i}=\varepsilon_{i}^{\alpha_{i}},\;i=1,2,\ldots ,N$$ (8)

$$Define:\;X_{q}\left(\varepsilon \right)=\sum_{i=1}^{N}P_{i}^{q}$$ (9)

where *X*_*q*_(*ε*) is the weight sum of probability of each area; *q* is the power of the probability density function *P*_*i*_ for the *i*_*th*_ area.

$$Define:\;D_{q}=\frac{1}{q-1}\underset{\varepsilon \to 0}{\lim}\frac{\ln X_{q}\left(\varepsilon \right)}{\ln\varepsilon }=\frac{1}{q-1}\underset{\varepsilon \to 0}{\lim}\frac{\ln\left(\sum_{i=1}^{N}P_{i}^{q}\right)}{\ln\varepsilon }$$ (10)

where *D*_*q*_ is the generalized multifractal dimensions.

From Eq (10), it can be seen that when *q*>>1, the areas with bigger probability play a major role in *X*_*q*_(*ε*), and *X*_*q*_(*ε*) and *D*_*q*_ mirror the characteristics of areas with bigger probability (i.e., areas with denseness). On the contrary, when *q*<<1, *X*_*q*_(*ε*) and *D*_*q*_ mirror the characteristics of areas with smaller probability (i.e., areas with sparseness). Therefore, the characteristics of different areas with different probability are reflected by different *q*, and a sequence of signals are divided into many areas, which are full of different singularity after weight sum of probability of each area. And thus the subtle structure of a sequence of signals can be described at different levels by the generalized multifractal dimensions.

## 3. Gray relation theory

After fault feature extraction, a fault pattern recognition technique is used to achieve automatically the rolling element bearing fault diagnosis. The study on gray relation theory is the foundation of gray system theory, which is based on the basic theory of space mathematics to calculate relation coefficient and relation degree between reference characteristic vectors and comparative characteristic vectors. Many investigations [29–32] have demonstrated that gray relation theory is full of capability to be used in machinery fault recognition with four reasons:

1. it has the ability to assist the selection of characteristic parameters for classification;
2. it has good tolerance to measurement noise;
3. it can solve the learning problem with a small number of samples;
4. its algorithm is simple and can alleviate the issue of generality versus accuracy.

Given the dominant characteristic vectors (i.e., the multifractal dimensions) of the fault features extracted from bearing vibration signals to be recognized is as follows.

$$B_{1}=\left[\begin{array}{l} D_{1,1}\\ D_{2,1}\\ \cdots \\ D_{K,1} \end{array}\right],\;B_{2}=\left[\begin{array}{l} D_{1,2}\\ D_{2,2}\\ \cdots \\ D_{K,2} \end{array}\right],\;\ldots ,\;B_{i}=\left[\begin{array}{l} D_{1,i}\\ D_{2,i}\\ \cdots \\ D_{K,i} \end{array}\right],\;\ldots$$ (11)

where *B*_*i*_(*i* = 1,2,…) is fault pattern to be identified; *D*_*k*,*i*_(*i* = 1,2,…) is each characteristic parameter; *K* is the total number of characteristic parameters selected as characteristic vector.

Consider the knowledge base from a small number of samples between fault signatures (i.e., the characteristic vectors) and fault patterns (i.e., the fault types of rolling element bearings as well as various levels of severity) is as follows.

$$C_{1}=\left[\begin{array}{l} c_{1}\left(1\right)\\ c_{1}\left(2\right)\\ \cdots \\ c_{1}\left(K\right) \end{array}\right],\;C_{2}=\left[\begin{array}{l} c_{2}\left(1\right)\\ c_{2}\left(2\right)\\ \cdots \\ c_{2}\left(K\right) \end{array}\right]\ldots ,\;C_{j}=\left[\begin{array}{l} c_{j}\left(1\right)\\ c_{j}\left(2\right)\\ \cdots \\ c_{j}\left(K\right) \end{array}\right],\;\ldots$$ (12)

where *C*_*j*_(*j* = 1,2,…) is a known fault pattern; *c*_*j*_(*j* = 1,2,…) is each characteristic parameter; *m* is the total number of fault patterns.

For *ρ* ∈ (0,1):

$$\xi \left(D_{k,i},c_{j}\left(k\right)\right)=\frac{\underset{j}{\min}\;\underset{k}{\min}\left|D_{k,i}-c_{j}\left(k\right)\right|+\rho \cdot \underset{j}{\max}\;\underset{k}{\max}\left|D_{k,i}-c_{j}\left(k\right)\right|}{\left|D_{k,i}-c_{j}\left(k\right)\right|+\rho \cdot \underset{j}{\max}\;\underset{k}{\max}\left|D_{k,i}-c_{j}\left(k\right)\right|}$$ (13)

$$\xi (B_{i},C_{j})=\frac{1}{K}\sum_{k=1}^{K}\xi \left(D_{k,i},c_{j}\left(k\right)\right),\;j=1,2,\ldots$$ (14)

where *ρ* is distinguishing coefficient, and its value is usually set as 0.5; *ξ*(*D*_*k*,*i*_,*c*_*j*_(*k*)) is the gray relation coefficient for *k*_th_ characteristic parameter of *B*_*i*_ and *C*_*j*_; *ξ*(*B*_*i*_,*C*_*j*_) is the gray relation degree of *B*_*i*_ and *C*_*j*_.

And then *B*_*i*_ is classified to the fault pattern in which the maximum *ξ*(*B*_*i*_,*C*_*j*_)(*j* = 1,2,…,) is obtained.

So as to enhance its tolerance to measurement noise and the ability to assist the selection of characteristic values for fault classification, the information theory was introduced into the calculation of the relation degree and that is so-called adaptive gray relation algorithm (GRA) [33].

First, process the distance of characteristic parameter |Δ*d*_*ij*_(*k*)| = |*D*_*k*,*i*_−*c*_*j*_(*k*)| and then calculate the probability as follows.

$$l_{ij}\left(k\right)=|\Delta d_{ij}\left(k\right)|/\sum_{j=1}^{M}\left|\Delta d_{ij}\left(k\right)\right|$$ (15)

where *M* is the total number of the known fault patterns in the knowledge base.

Define the entropy value as follows.

$$E_{ij}(k)=-\sum_{j=1}^{M}l_{ij}\left(k\right)\ln l_{ij}\left(k\right)$$ (16)

And the maximum entropy value is as follows.

$$E_{\max}={\left[-\sum_{j=1}^{M}l_{ij}\left(k\right)\ln l_{ij}\left(k\right)\right]}_{\max}=-\sum_{i=1}^{M}\frac{1}{M}\ln\frac{1}{M}=\ln M$$ (17)

Then, the relative entropy value is obtained as follows.

$$e_{ij}(k)=E_{ij}(k)/E_{\max}$$ (18)

Referred to the concept of surplus degree in information theory, the definition of surplus degree for *k*_th_ characteristic parameter is as follows.

$$H_{ij}(k)=1-e_{ij}(k)$$ (19)

The essential meaning of surplus degree is to remove the difference between the entropy value of the *k*_th_ characteristic parameter and the optimal entropy value of characteristic parameter. The bigger *H*_*ij*_(*k*) is, the more important the *k*_th_ characteristic parameter is, and then the *H*_*ij*_(*k*) should be granted greater weight.

Finally, calculate the weight coefficient *a*_*ij*_(*k*) for *k*_th_ characteristic parameter as follows.

$$a_{ij}(k)=H_{ij}(k)/\sum_{k=1}^{K}H_{ij}(k)$$ (20)

where $\sum_{k=1}^{K}a_{ij}(k)=1$, *a*_*ij*_(*k*) ≥ 0.

Then obtain relation degree by weight coefficient multiplying with the corresponding relation coefficient as follows.

$$\xi (B_{i},C_{j})=\frac{1}{K}\sum_{k=1}^{K}a_{ij}(k)\cdot \xi \left(D_{k,i},c_{j}\left(k\right)\right)$$ (21)

And finally *B*_*i*_ is classified to the fault pattern in which the maximum *ξ*(*B*_*i*_,*C*_*j*_)(*j* = 1,2,…,*M*) is obtained, i.e., the the fault types of rolling element bearings as well as various levels of severity are recognized.

## 4. Proposed Approach

The extraction of subtle features from bearing vibration signals is the core of a rolling element bearing fault diagnosis, and its quality will directly produce essential effect on the success rate of the subsequent fault pattern recognition. With the integration of the advantages of multifractal dimension algorithm and adaptive gray relation algorithm, a novel fault diagnosis method for rolling element bearings is put forward as follows.

1. The bearing vibration signals under different health status are acquired as samples which are divided into two subsets, the samples for knowledge base and the samples for testing.
2. The dominant characteristic vectors of fault features from the bearing vibration signals, which can provide more useful information concerning bearing health status, were extracted by multifractal dimension algorithm.
3. Establish knowledge base according to the relationship between fault signatures (i.e., the characteristic vectors) and fault patterns (i.e., the fault types of rolling element bearings as well as different levels of severity) from the base samples for adaptive GRA model.
4. The feature vectors of the testing samples are input into the adaptive GRA model, and various bearing health status can be recognized by the output of the adaptive GRA model.

**Fig 1:**
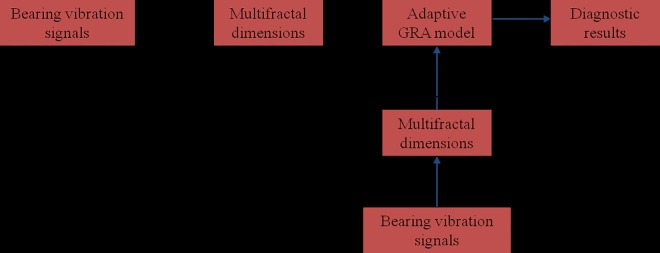
A rolling element bearing fault diagnostic system based on multifractal dimension algorithm and adaptive gray relation algorithm

And the diagnostic procedure is shown in Fig 1.

The detailed procedure of the proposed method is as follows:

1) Discretize the unknown received bearing vibration signals:

Suppose the received bearing vibration signals is *s*, and the sequence of preprocessed discrete bearing vibration signals is {*s*(*i*)}, *i* = 1,2,⋯,*N*_0_, where *N*_0_ is the length of the sequence.

2) Recombine the sequence of discrete bearing vibration signals {*s*(*i*)}, *i* = 1,2,⋯,*N*_0_:

$$Define:\;n=\log_{2}N_{0}$$ (22)

where *n* is the time of recombining the sequence of discrete bearing vibration signals.

$$Define:\;t\left(j\right)=2^{j},\;j=1,2,\ldots ,n$$ (23)

where *t*(*j*) is the number of discrete bearing vibration signal points for *j*_*th*_ recombination.

$$Define\;sequence:\;T\left(j\right)=\frac{N_{0}}{t\left(j\right)}=\frac{N_{0}}{2^{j}},\;j=1,2,\ldots ,n$$ (24)

And then define the sequence of discrete bearing vibration signals *S*(*j*) for the *j*_*th*_ recombination:

$$S\left(j\right)=s\left(T\left(j\right)\cdot \left(t\left(j\right)-1\right)+T_{0}\left(j\right)\right)$$ (25)

where *T*_0_(*j*) = [1:*T*(*j*)], *j* = 1,2,…,*n*.

3) Extract multifractal dimensions from the total sequence of recombined discrete bearing vibration signals *S*(*j*), *j* = 1,2,⋯,*n*:

Divide the research object (i.e., the total sequences of recombined discrete bearing vibration signals *S*(*j*), *j* = 1,2,⋯,*n*) into *N* number of small areas respectively, and consider the linear dimension of the *i*_th_ area is *ε*_*i*_, and thus the probability density function *P*_*i*_ for the *i*_th_ area with different scale index *α*_*i*_ can be described as:

$$P_{i}=\varepsilon_{i}^{\alpha_{i}}\;,\;i=1,2,\cdots ,N$$ (26)

where *α*_*i*_ is singular index and is the fractal dimension for *i*_th_ area. As the total sequences of recombined discrete bearing vibration signals *S*(*j*), *j* = 1,2,⋯,*n*, has been divided into *N* number of small areas, thus a series of *α*_*i*_ (*i* = 1,2,⋯,*N*) can be obtained and made up of a variable *f*(*α*) which is the multifractal spectrum for each sequence of recombined discrete bearing vibration signals.

$$Define:\;X_{q}\left(\varepsilon \right)=\sum_{i=1}^{N}P_{i}^{q}$$ (27)

where *X*_*q*_(*ε*) is the weight sum of probability of each area; *q* is the power of the probability density function *P*_*i*_ for the *i*_*th*_ area.

$$Define:\;D_{q}=\frac{1}{q-1}\underset{\varepsilon \to 0}{\lim}\frac{\ln X_{q}\left(\varepsilon \right)}{\ln\varepsilon }=\frac{1}{q-1}\underset{\varepsilon \to 0}{\lim}\frac{\ln\left(\sum_{i=1}^{N}P_{i}^{q}\right)}{\ln\varepsilon }$$ (28)

where *D*_*q*_ is a generalized multifractal dimension.

Obtain the sum *S*_*j*_ of the *j*_*th*_ sequence of recombined discrete bearing vibration signals *S*(*j*):

$$\begin{array}{l} S_{j}=\sum S\left(j\right)=\sum s\left(T\left(j\right)\cdot [t\left(j\right)-1]+T_{0}\left(j\right)\right)\\ \;=\sum_{T_{0}\left(j\right)=1}^{T\left(j\right)}s\left(T\left(j\right)\cdot [t\left(j\right)-1]+T_{0}\left(j\right)\right) \end{array}$$ (29)

Obtain the sum *S* of the whole discrete bearing vibration signals {*s*(*i*)}, *i* = 1,2,…,*N*_0_:

$$S=\sum_{i=1}^{N_{0}}s\left(i\right)$$ (30)

And then calculate probability density function *P*_*j*_ for the *j*_th_ area:

$$P_{j}=\frac{S_{j}}{S},\;j=1,2,\cdots ,n$$ (31)

The generalized multifractal dimension *D*_*q*_ can be obtained by inputting Eq (31) to Eq (28).

4) Establish knowledge base for adaptive GRA model and various unknown bearing health status can be recognized by the output of the adaptive GRA model:

Set *q* as from −*q*_0_ to *q*_0_, and then there are 2*q*_0_ + 1 sets of multifractal dimensions, in which there are *n* = log_2_ *N*_0_ number of characteristic points for each *q*. Thus the total number of characteristic parameters for a sequence of preprocessed discrete bearing vibration signals {*s*(*i*)}, *i* = 1,2,…,*N*_0_, is *M* = (2*q*_0_ + 1)·*n* = (2*q*_0_ + 1)·log_2_ *N*_0_, to constitute one set of characteristic vector. And finally the unknown bearing health status can be classified to the fault pattern in which the maximum relation degree is obtained, i.e., the the fault types of rolling element bearing as well as various levels of severity is recognized by Eq (15) to Eq (21).

## 5. Experimental Validation

### 5.1 Experimental rig

**Fig 2:**
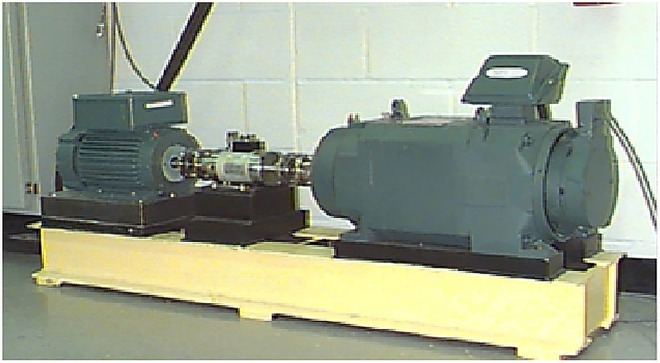
Experimental rig

All the rolling element bearing vibration signals used for analysis were downloaded from Case Western Reserve University Bearing Data Center [34]. The deep groove balling bearing 6205-2RS JEM SKF was used in the experiment. The whole experimental rig consists of a two horsepower three-phase induction motor, a torque transducer and a dynamometer, as shown in Fig 2.

The desired torque load levels could be achieved by controlling the dynamometer. The horsepower and speed data are collected by the sensor. The motor shaft at the drive end is supported by the test bearing, and an accelerometer with a bandwidth up to 5000Hz was installed on the motor housing at the drive end of the motor, and then bearing vibration data under different working conditions were collected using a recorder with a sampling frequency of 12 kHz. Here single point faults were introduced into the test bearing by using electro-discharge machining, with various fault diameters of 7mils, 14mils, 21mils, and 28mils. Rolling bearing faults under consideration include inner race fault, ball fault and outer race fault.

### 5.2 Application and analysis

To assess the effectiveness of the proposed method, experimental analyses of rolling element bearing fault diagnosis were carried out. The bearing vibration signals used for the analyses were obtained from the tests which were conducted at the load of 0 horsepower and at the motor speed of 1797 r/min. Normal and three fault types of bearing vibration data as well as those with various severities for each fault type are analyzed, and detailed description of the related vibration data was shown in Table 1. Considering various fault categories and severities, the rolling element bearing fault diagnosis turns out to be an 11-class fault pattern recognition problem. The data set was made up of totally 550 data samples, in which each data sample was cut into a 2048-point time series from the original vibration data and there is no overlap between any two of them. Among these 550 data samples, 110 samples were selected at random as the samples for knowledge base, and the rest 440 were automatically treated as testing data. Here the testing data are four times larger than the amount of the training data, as the bearing vibration data under faulty conditions is hard to obtain and is of small sample size as usual in the practical applications.

**Table 1:** Description of experimental data set

Bearing condition	Fault size (mils)	The number of base samples	The number of testing samples	Label of classification
Normal	0	10	40	1
Inner race fault	7	10	40	2
	14	10	40	3
	21	10	40	4
	28	10	40	5
Ball fault	7	10	40	6
	14	10	40	7
	28	10	40	8
Outer race fault	7	10	40	9
	14	10	40	10
	21	10	40	11

**Fig 3:**
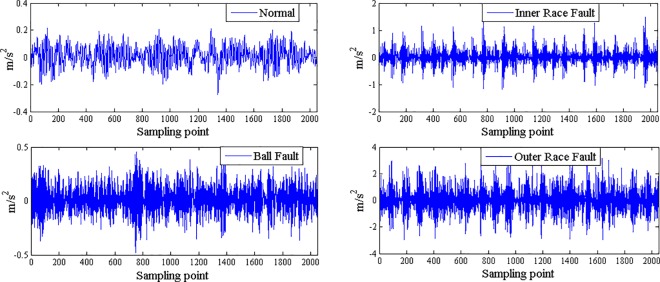
Bearing normal condition and various fault conditions with fault size 7mils

**Fig 4:**
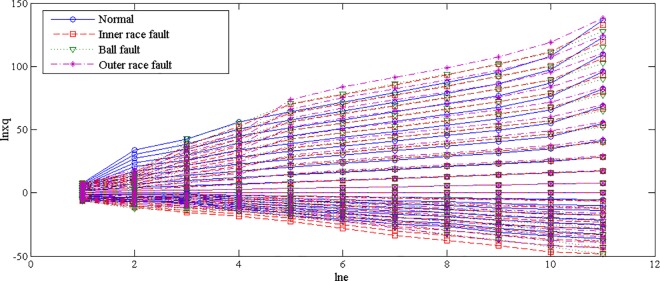
Generalized multifractal dimensions of a random chosen sample from bearing normal condition and different fault conditions with fault size 7mils

**Fig 5:**
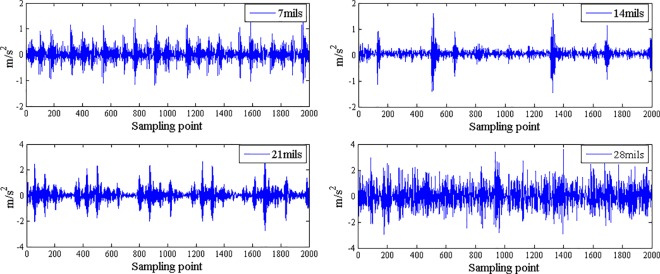
Bearing inner race fault conditions with different levels of severity.

The fault features extracted from bearing normal condition and various fault conditions with fault size 7mils (seen Fig 3) by traditional single fractal dimension (i.e., box-counting dimension) was shown in Table 2 and by generalized multifractal dimensions were shown in Fig 4. And the fault features extracted from bearing inner race fault condition with different levels of severity (seen Fig 5) by single fractal dimension (i.e., box-counting dimension) was shown in Table 3 and by generalized multifractal dimensions were shown in Fig 4.

**Table 2:** Traditional single fractal dimension (i.e., box-counting dimension) of a random chosen sample from bearing normal condition and different fault conditions with fault size 7mils

Signals	Normal	Inner race fault	Ball fault	Out race fault
Traditional box-counting dimension	1.5718	1.6173	1.7511	1.6000

**Table 3:** Traditional single fractal dimension (i.e., box-counting dimension) of a random chosen sample from bearing inner race fault condition with different levels of severity

Signals	7mils	14mils	21mils	28mils
Traditional box-counting dimension	1.6173	1.5795	1.6356	1.6491

Note: In Figs 4 and 6, the abscissa axis represents the dimensions of reconstructed characteristic vector space, denoted as “lne”, and the ordinate axis represents $\ln\left(\sum_{i=1}^{N}P_{i}^{q}\right)$, denoted as “lnXq”.

**Fig 6:**
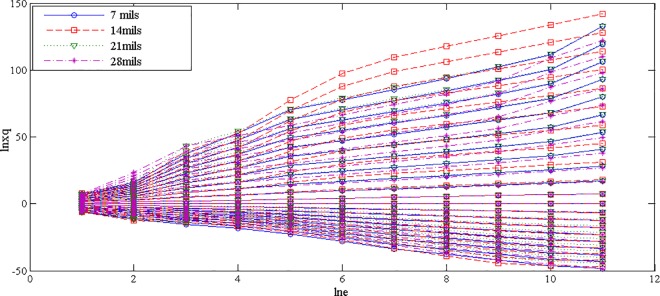
Generalized multifractal dimensions of a random chosen sample from bearing inner race fault condition with different levels of severity

From Tables 2 and 3, it is can be seen that the fault features extracted by traditional single fractal dimension (i.e., box-counting dimension) method are limited, and the distances between various fault types of rolling element bearing as well as various levels of severity are close.

From Figs 4 and 6, it is interesting to see that the dominant characteristic vectors of fault features extracted from the rolling element bearing vibration signals with different fault types as well as various levels of severity by the generalized multifractal dimensions are rich.

After establishing knowledge base according to the relationship between fault signatures (i.e., the characteristic vectors) and fault patterns (i.e., the fault types of rolling element bearings as well as different levels of severity) from the base samples for adaptive GRA model, the characteristic vectors of the testing samples were input into the adaptive GRA model, and various bearing health status were recognized by the output of the adaptive GRA model shown in Table 4.

**Table 4:** The fault pattern recognition results with traditional single fractal dimension (i.e., box-counting dimension) and generalized multifractal dimensions

Label of classification	The number of testing samples	The number of misclassified samples		Testing accuracy (%)	
		Traditional	Generalized	Traditional	Generalized
1	40	18	0	55	100
2	40	8	0	80	100
3	40	20	0	50	100
4	40	18	3	55	92.5
5	40	14	0	65	100
6	40	22	2	45	95
7	40	31	3	22.5	92.5
8	40	32	3	20	92.5
9	40	22	0	45	100
10	40	31	0	22.5	100
11	40	16	4	60	90
In total	440	232	15	47.2727	96.59

From Table 4, the identification results show that the recognition effect with traditional single fractal dimension (i.e., box-counting dimension) is poor and misleading due to the limited fault features extracted, while the recognition effect with generalized multifractal dimensions seems much better, in which the recognition success rate for detecting bearing faulty conditions is 100% and the total success rate is 96.59% for identifying different bearing fault types as well as severities.

### 5.3 Further discussion

To further analyze the effectiveness of the proposed method for dealing with the learning problem with an extremely small number of samples, another experimental test in which only one random base sample for these 11 classifications used for establishing knowledge base for the adaptive GRA model was carried out. The feature vectors of the testing samples were also input into the adaptive GRA model, and various bearing health status were recognized by the output of the adaptive GRA model shown in Table 5.

**Table 5:** The fault pattern recognition results

Label of classification	The number of testing samples	The number of misclassified samples		Testing accuracy (%)	
		Traditional	Generalized	Traditional	Generalized
1	40	38	0	5	100
2	40	7	0	82.5	100
3	40	27	2	30	95
4	40	29	15	27.5	62.5
5	40	6	11	85	72.5
6	40	27	17	32.5	57.5
7	40	28	6	30	85
8	40	23	22	42.5	45
9	40	10	0	75	100
10	40	33	0	17.5	100
11	40	17	10	57.5	75
In total	440	245	83	44.0909	81.14

From Table 5, it is encouraging to see that the proposed method shows good robust recognition effect over this extreme experimental validation and the total success rate can still reach more than 81%, while the recognition success rate for detecting bearing faulty conditions is still 100%.

In the future research, for improving the diagnostic accuracy of the proposed approach, other advanced signal processing methods (e.g., wavelet package transform (WPT), hilbert transform (HT), empirical mode decomposition (EMD) or higher order spectra (HOS)), should be explored to be integrated into the generalized multifractal dimensions to more effectively extract dominant characteristic vectors.

## 6. Conclusion

Rolling element bearings as an important component in almost all types of rotating machines have been widespreadly used and its failure is one of the foremost causes of failure and breakdowns in rotating machinery, resulting in significant economic loss. In the paper, a novel approach for rolling element bearing fault diagnosis was proposed based on generalized multifractal dimension algorithm and adaptive gray relation algorithm. First, the fault features from the bearing vibration signals, which can provide more useful information reflecting bearing health status were extracted by the generalized multifractal dimension algorithm. And then, the fault types of rolling element bearings as well as different levels of severity are recognized by the outputs of the adaptive GRA algorithm. The experimental results demonstrate that the proposed method can effectively and accurately identify different bearing fault types as well as severities. And some other meaningful conclusions can be obtained as follows:

1. Traditional single fractal dimension is not enough to describe the rolling element bearing health status, while the multifractal dimensions are suitable to be applied to extract fault features from the rolling element bearing vibration signals, which offer more meaningful and distinguishing information reflecting different bearing health status.
2. Gray relation theory is full of capability to be used in the rolling element bearing fault pattern recognition, and the recognition success rate for detecting bearing faulty condtions is 100% and the total success rate can be more than 96% for identifying different bearing fault types as well as severities by the proposed method.
3. When the number of samples for knowledge base becomes extremely limited, the total success rate may be reduced significantly for identifying different bearing fault types as well as severities, while the recognition success rate for detecting bearing faulty conditions is still 100%.
